# Patient safety culture in Italian out-of-hours primary care service: a national cross-sectional survey study

**DOI:** 10.3399/bjgpopen20X101098

**Published:** 2020-11-11

**Authors:** Jacopo Demurtas, Pierpaolo Marchetti, Alberto Vaona, Nicola Veronese, Stefano Celotto, Ellen Catharina Deilkås, Dag Hofoss, Gunnar Tschudi Bondevik

**Affiliations:** 1 OOH Primary Care Service, Azienda USL Sud Est Toscana, Grosseto, Italy; 2 Clinical and Experimental Medicine PhD Program, University of Modena and Reggio Emilia, Modena, Italy; 3 Department of Diagnostics and Public Health, Verona University, Verona, Italy; 4 OOH Primary Care Service, Azienda ULSS 9 Scaligera, Verona, Italy; 5 Primary Care Department, Azienda ULSS 3 Serenissima, Venice, Italy; 6 Primary Care Department, Azienda Sanitaria Universitaria Friuli Centrale, Udine, Italy; 7 Health Services Research Unit, Akershus University Hospital, Lørenskog, Norway; 8 Department of Postgraduate Studies, Lovisenberg Diaconal University College, Oslo, Norway; 9 National Centre for Emergency Primary Health Care, NORCE Norwegian Research Centre, Bergen, Norway; 10 Department of Global Public Health and Primary Care, University of Bergen, Bergen, Norway

**Keywords:** Healthcare quality improvement, safety management, patient safety, statistical factor analysis, Italy, surveys and questionnaires

## Abstract

**Background:**

Out-of-hours (OOH) services in Italy provide >10 million consultations every year. To the authors' knowledge, no data on patient safety culture (PSC) have been reported.

**Aim:**

To assess PSC in the Italian OOH setting.

**Design & setting:**

National cross-sectional survey using the Safety Attitudes Questionnaire — Ambulatory Version (SAQ-AV).

**Method:**

The SAQ-AV was translated into Italian and distributed in a convenience sample of OOH doctors in 2015. Answers were collected anonymously by Qualtrics. Stata (version 14) was used to estimate Cronbach’s alpha, perform exploratory and confirmatory factor analysis, correlate items to doctors’ characteristics, and to do item descriptive analysis.

**Results:**

Overall, 692 OOH doctors were contacted, with a 71% response rate. In the exploratory factor analysis (EFA), four factors were identified: Communication and Safety Climate (14 items); Perceptions of Management (eight items); Workload and Clinical Risk (six items); and Burnout Risk (four items).

These four factors accounted for 68% of the total variance (Kaiser–Meyer–Olkin [KMO] statistic = 0.843). Cronbach’s alpha ranged from 0.710–0.917. OOH doctors were often dissatisfied with their job; there is insufficient staff to provide optimal care and there is no training or supervision for new personnel and family medicine trainees. Service managers are perceived as distant, with particular issues concerning the communication between managers and OOH doctors. A large proportion of OOH doctors (56.8%) state that they do not receive adequate support.

**Conclusion:**

These findings could be useful for informing policies on how to improve PSC in Italian OOH service.

## How this fits in

Patient safety culture (PSC) is an insufficiently studied topic in Italy. A number of reports in hospital settings exist but, to the authors' knowledge, PSC has not been assessed in Italy in outpatient settings. This study could help inform policies to strengthen PSC in out-of-hours (OOH) service and improve communication between directors and doctors, leading to better health care.

## Introduction

The Italian OOH service provides >10 million consultations every year and ensures care for patients with urgent healthcare needs.^1^ Safe, good, consistent, and effective OOH primary care services are crucial for providing care as close to a patient’s home as possible.^1–4^

PSC is how leaders and staff interactions, attitudes, routines, and practices protect patients from adverse events in health care.^3^ PSC, therefore, should be considered as a group phenomenon rather than a phenomenon regarding individuals.^3,5^ Several organisations, such as the UK NHS,^1^ the Joint Commission for the Accreditation of Healthcare Organisations, the Agency for Healthcare Research and Quality,^6^ and the US National Quality Forum encourage measurements of safety culture. Safety attitude evaluation, born in aviation and in the military field,^7,8^ was the model for patient safety attitudes of healthcare providers^2^ and for the Safety Attitude Questionnaire (SAQ),^9,10^ widely used in hospital settings and then adapted to outpatient primary care setting in an SAQ-Ambulatory Version (SAQ-AV).^2,11,12^

Research on PSC began in hospitals and is still focused on secondary care. Nevertheless, the bulk of services are outside of hospitals. As Italian OOH services deliver millions of consultations every year, it is important to expand the scope of PSC research to also include this aspect of health care. The SAQ-AV has been recently used as part of the SAFE-EUR-OOH project in Norway,^3^ the Netherlands,^11^ and Slovenia^5^ to measure PSC. In these surveys, the questions relevant to describing a single dimension of professional healthcare activity and setting which may impact on patient safety are aggregated into clusters called factors. To the best of the authors' knowledge, there have been no studies on PSC in Italian OOH services. The aims of this study were to translate the SAQ-AV into Italian, to test the reliability of the factor structure, and to assess whether the factors were related to demographic characteristics in Italian OOH services.

## Method

### Type of study and setting

Doctors working in OOH services across all of the Italian regions divided into three geographical areas (North, Centre, South and Islands) were involved in a cross-sectional study. Doctors without an employment contract with the Local Health Trust were excluded.

### Research instrument

The original SAQ-AV was translated from English into Italian according to modified principles from Beaton,^13^ which allow a cross-cultural adaptation of the contents of a questionnaire assuring a prompt understanding of the surveys by readers. The SAQ-AV consists of 62 items, all rated on a 5-point Likert scale by which the responders indicate their level of agreement with the statement (1 = disagree strongly, 2 = disagree slightly, 3 = neutral, 4 = agree slightly, 5 = agree strongly). In the analysis, scores of negatively worded items were reversed, so that higher scores in the dataset always indicated a more positive evaluation of the PSC. For all questions, 'Not Applicable' was included as a response category and was combined with missing values in the data analyses.

Demographic variables measured were: sex (male, female), age (≤30, 31–40, 41–50, >50 years), position (medical doctors/interns, managers), working experience (≤5, 6–10, 11–20, >20 years), years in this workplace (≤2, 3–5, 6–10, 11–20, >20 years), work shift (diurnal/evening, night, mixed), and employment contract (full-time or part-time contract work).

### Data collection

On 23 March 2015, the research team used the data collection program Qualtric to send the survey to all the participants, from the coordinating centre in Norway. Automatic reminders were sent after 1 week and 1 month to those who had not answered, and time was given to fill in the questionnaire up until 6 May 2015. All questions were obligatory to answer. The period of data collection lasted 54 days. OOH doctors were recruited in collaboration and agreement with two primary care doctors’ organisations: *Federazione Italiana Medici di Medicina Generale*, the principal union of GPs in Italy, and *Movimento Giotto*, the cultural movement of young GPs in Italy, which spread the invitation to receive the SAQ-AV by email.

The electronic questionnaire was sent to 692 enrolled OOH doctors who had expressed their interest in taking part in the survey. Of these, 491 subjects completed the questionnaire (response rate 71%). Thirty-three subjects were excluded because all variables (demographic characteristics and questionnaire) were missing. To perform the Exploratory Factor Analysis (EFA), items were excluded whose missing counts exceeded 20% (Supplementary Table S1 and Table S2) and subjects who had <75% of valid answers to items (*n* = 49). The final number of subjects included in the analysis was 409.

### Statistical analysis

The Qualtrics files with anonymous SAQ-AV data were used to estimate the Cronbach’s alphas, item-to-own-factor correlations, intercorrelations of factors, and item-descriptive statistics. The original SAQ, developed at the University of Texas at Austin, described six factors: Teamwork climate, Safety climate, Working conditions, Job satisfaction, Perceptions of management, and Stress recognition. Since several studies have found that the factor Stress recognition did not vary significantly among organisational units^14^ and also had problems regarding construct validity , it cannot be considered a valid factor for measuring patient safety . Moreover, items originally included in two different factors (Safety climate and Teamwork climate) presented similar loadings, and were therefore clustered together in the analysis. Hence, the hypothesised factor model included four factors.

The factors reflect the correlation structure in the item responses. Valid factors should reflect a thematic logic that is coherent with the purpose of the questionnaire. The hypothesised model was tested by Confirmatory Factor Analysis (CFA) and the fit of the model was described by four goodness of fit indicators: the Χ^2^ test, the p_close_ value, the comparative fit index (CFI), the root mean squared error of approximation (RMSEA), and the Tucker–Lewis index (TLI).^15,16^

Internal consistency (reliability) was measured by Cronbach’s alpha and associations between the factors were assessed by Pearson’s *r*. For each factor, the mean and standard deviation was computed for all items included in the factor.

Categorical demographic variables were summarised as frequencies and percentages. To compare mean factor scores in the demographic characteristics, the Student *t*-test or analysis of variance (ANOVA) was used when the variable had at least two groups. A *P* value <0.05 was considered statistically significant. Statistical analyses were performed with Stata (version 14).

## Results

The surveyed physicians came mainly from Northern Italy (North 48.7%, Centre 14.9%, South and Islands 36.4%). Approximately one-third (37.4%) of them were aged 31–40 years, and slightly more than half were female (52.4%). Most of the physicians had been working for <10 years (56.5%), and almost 40% at the current place of work for <2 years.

### Statistics

The EFA included the 172 subjects who had answered each of the items, meaning every single SAQ question, or the subset the authors decided to analyse by EFA (no Missing or Not Applicable data). Four factors were extracted:

- Communication and Safety Climate (14 items);
- Perceptions of Management (eight items);
- Workload and Clinical Risk (six items); and
- Burnout Risk (four items).

Therefore, there were 32 items in total (Table 1).

**Table 1.** Factor loadings of the 32 items in the four factor structure

**Item**	**Factor 1**
45^†^. Attending physicians/primary care providers in this office are doing a good job	0.714
51. There is widespread adherence to clinical guidelines and evidence-based criteria in this office	0.651
05. Medical errors* are handled appropriately in this office	0.636
48. Patient safety is constantly reinforced as the priority in this office	0.616
30. Disagreements in this office are resolved appropriately (that is, not *who* is right but *what* is best for the patient)	0.614
15. This office is a good place to work	0.593
08. Working in this office is like being part of a large family	0.582
29. I am proud to work at this office	0.579
41. Morale in this office is high	0.576
50. Important issues are well communicated at shift changes	0.564
21. The culture in this office makes it easy to learn from the errors of others	0.559
04. I would feel safe being treated here as a patient	0.548
46. All the personnel in this office take responsibility for patient safety	0.547
20. I am encouraged by my colleagues to report any patient safety concerns I may have	0.538
	**Factor 2**
10. The management of this office supports my daily efforts	0.739
09. Senior management of this office is doing a good job	0.664
26. I am provided with adequate, timely information about events in the office that might affect my work	0.642
14. Briefings are common in this office	0.597
28. I know the proper channels to direct questions regarding patient safety in this office	0.584
11. I receive appropriate feedback about my performance	0.532
06. This office does a good job of training new personnel	0.523
23. The medical equipment is this office is adequate	0.515
	**Factor 3**
44. I have made errors that had the potential to harm patients	0.577
33. Stress from personal problems adversely affects my performance	–0.604
56. Fatigue impairs my performance during emergency situations (for example, code or cardiac arrest)	–0.617
31. I am less effective at work when fatigued	–0.651
57. Fatigue impairs my performance during routine care	–0.663
32. I am more likely to make errors in tense or hostile situations	–0.703
	**Factor 4**
49. I feel burned out from my work*	0.642
53. I feel I am working too hard on my job*	0.609
52. I feel frustrated by my job*	0.597
01. High levels of workload are common in this office*	0.543

Factor 1 = Communication and Safety Climate. Factor 2 = Perceptions of Management. Factor 3 = Workload and Clinical Risk. Factor 4 = Burnout Risk.

* reverse item

† numbers indicate the original SAQ-AV question number

Table 2 shows the results of the CFA based on the factors extracted in the EFA. The model’s *P* value was <0.001, but the other indices showed an acceptable fit (CFI = 0.815, TLI = 0.799, RMSEA = 0.077). The KMO statistic was 0.843 and the Cronbach’s alphas ranged from 0.710–0.917, indicating an acceptable reliability. The four factors accounted for 68% of the total variance.

**Table 2.** Results of CFA

**Factor**	**Χ^2^**	***P* value**	**RMSEA**	**p close**	**CFI**	**TLI**	**Cronbach's alpha**
1 Communication and Safety Climate	328	<0.001	0.102	<0.001	0.884	0.862	0.917
2 Perceptions of Management	86	<0.001	0.102	<0.001	0.927	0.898	0.858
3 Workload and Clinical Risk	84	<0.001	0.155	<0.001	0.887	0.811	0.797
4 Burnout Risk	18	<0.001	0.141	0.004	0.960	0.881	0.710
Whole model	1078	<0.001	0.077	<0.001	0.815	0.799	

*P* should exceed 0.05. CFI should be close to 1. RMSEA should not exceed 0.10. TLI should be close to 1.

CFI comparative fit index

RMSEA root mean square error of approximation CFA confirmatory factor analysis TLI Tucker–Lewis index

The Pearson’s correlation (Table 3) was positive between the first and the second factor (*r* = 0.64, *P*<0.001), while the third factor showed a negative correlation with all three factors (*r* = −0.25 with factor one, *r* = −0.17 with factor two, *r* = −0.33 with factor three; all correlations had *P*<0.001).

**Table 3.** Mean, SD score, and intercorrelations of the factors

	**Mean**	SD	**Factor 1**	**Factor 2**	**Factor 3**	**Factor 4**
Factor 1 Communication and Safety Climate	3.35	0.84	1.00			
Factor 2 Perceptions of Management	2.25	0.85	0.64	1.00		
Factor 3 Workload and Clinical Risk	2.14	0.89	–0.25	–0.17	1.00	
Factor 4 Burnout Risk	3.05	0.93	0.26	0.18	–0.33	1.00

All correlations were significant (*P*<0.001)

SD standard deviation

The mean score in each factor was analysed (Table 4). For Communication and Safety Climate, the males’ mean score was significantly higher than the females’ mean score (3.47±0.79 versus 3.22±0.87; *P* = 0.002). Subjects in the 31–40 age group had a significantly lower factor mean score than younger and older subjects (3.07±0.81 in 31–40 years versus 3.56±0.74 in ≤30 years and 3.51±0.88 in >50 years; *P*<0.001). Subjects with more years of working experience had a significantly higher mean score than those with less experience (*P* = 0.01) and the mean scores increased significantly with the years spent at the same workplace (*P*<0.001).

**Table 4.** Proportion of agreement and mean scores of each item in factor structure

**Factors and items**	**Agreement** ^a^ **(%**)	**Mean (**SD)
F1. Communication and Safety Climate		
45.Attending physicians/primary care providers in this office are doing a good job	77.5	4.0 (0.9)
50.Important issues are well communicated at shift changes	74	3.9 (1.2)
04.I would feel safe being treated here as a patient	70.6	3.8 (1.0)
15.This office is a good place to work	62.4	3.5 (1.1)
46.All the personnel in this office take responsibility for patient safety	60.7	3.6 (1.2)
29.I am proud to work at this office	53.1	3.4 (1.2)
05.Medical errors are handled appropriately in this office	48.6	3.3 (1.1)
51.There is widespread adherence to clinical guidelines and evidence-based criteria in this office	47.4	3.2 (1.2)
30.Disagreements in this office are resolved appropriately (that is, not *who* is right but *what* is best for the patient)	46.6	3.1 (1.3)
21.The culture in this office makes it easy to learn from the errors of others	42.9	3.0 (1.2)
48.Patient safety is constantly reinforced as the priority in this office	42.4	3.1 (1.4)
08.Working in this office is like being part of a large family	41.2	2.8 (1.4)
41.Morale in this office is high	40.8	3.1 (1.2)
20.I am encouraged by my colleagues to report any patient safety concerns I may have	38.2	3.0 (1.4)
		
**F2. Perceptions of Management**		
28.I know the proper channels to direct questions regarding patient safety in this office	39.1	2.8 (1.4)
26.I am provided with adequate, timely information about events in the office that might affect my work	25.1	2.4 (1.3)
06.This office does a good job of training new personnel	23.1	2.3 (1.3)
09.Senior management of this office is doing a good job	19.1	2.4 (1.2)
23.The medical equipment is this office is adequate	18.6	2.1 (1.2)
11.I receive appropriate feedback about my performance	18.4	2.2 (1.2)
10.The management of this office supports my daily efforts	17.7	2.2 (1.2)
14.Briefings are common in this office	14.3	1.9 (1.2)
		
**F3. Workload and Clinical Risk**		
31.I am less effective at work when fatigued	84.1	4.2 (1.0)
32.I am more likely to make errors in tense or hostile situations	62.7	3.6 (1.3)
44.I have made errors that had the potential to harm patients	55.9	3.7 (1.3)
57.Fatigue impairs my performance during routine care	46.3	3.0 (1.3)
56.Fatigue impairs my performance during emergency situations (for example, code or cardiac arrest)	46.2	3.0 (1.4)
33.Stress from personal problems adversely affects my performance	33.5	2.7 (1.3)
		
**F4. Burnout Risk**		
52.I feel frustrated by my job	60.6	3.7 (1.4)
49.I feel burned out from my work	51.6	3.4 (1.3)
53.I feel I am working too hard on my job	40.4	3.1 (1.3)
01.High levels of workload are common in this office	11.5	2.0 (1.1)

a Possible answers: agree slightly/agree strongly.

SD standard deviation

Perceptions of Management showed similar results to those of the first factor. The score for males was higher than that for females (2.41±0.88 versus 2.20±0.88; *P* = 0.02). Subjects in the 31–40 age group had a significantly lower factor mean score than younger and older subjects (2.14±0.78 in 31–40 years versus 2.54±0.80 in ≤30 years, and 2.38±0.99 in >50 years; *P* = 0.02). Subjects with >20 years work in the same clinic had a higher mean score than subjects working fewer years (*P* = 0.03). For Burnout Risk the mean score was also higher in males than females (3.14±0.94 versus 2.96±0.91; *P* = 0.05). No significant differences were found in demographic variables in Workload and Clinical Risk. The variables occupation, work shift, and employment contract showed no significant differences for any factor.

In the Communication and Safety Climate and Perceptions of Management factors, the mean scores increased significantly with the years worked at the workplace (*P*<0.001 and *P* = 0.03, respectively). For the variables occupation, work shift, and employment contract, no significant differences were found for any factor (Table 5).

**Table 5.** Mean score of factors and demographic characteristics

			**F1**(**Communication and Safety Climate**)	**F2**(**Perceptions of** **Management**)	**F3**(**Workload and Clinical Risk**)	**F4**(**Burnout Risk**)
**Variables**	*N*	**%**	**mean±SD**	***P*^a^**	**mean±SD**	***P*^a^**	**mean±SD**	***P*^a^**	**mean±SD**	***P*^a^**
**Occupation**				0.82		0.98		0.13		0.50
Medical doctors/interns	400	97.8	3.34±0.85		2.30±0.88		3.37±0.73		3.04±0.93	
Managers	9	2.2	3.27±0.68		2.31±1.03		3.74±0.66		3.25±0.71	
**Sex**				0.002		0.02		0.51		0.05
Male	196	47.9	3.47±0.79		2.41±0.88		3.35±0.76		3.14±0.94	
Female	213	52.1	3.22±0.87		2.20±0.88		3.40±0.71		2.96±0.91	
**Age, years**				<0.001		0.02		0.52		0.20
≤30	52	12.7	3.56±0.74		2.54±0.80		3.26±0.78		3.28±0.83	
31–40	153	37.4	3.07±0.81		2.14±0.78		3.40±0.63		3.03±0.91	
41–50	80	19.6	3.44±0.78		2.32±0.93		3.44±0.70		3.06±0.92	
>50	124	30.3	3.51±0.88		2.38±0.99		3.34±0.84		2.95±0.99	
**Working experience, years**				0.01		0.54		0.37		0.61
≤5	140	34.2	3.24±0.82		2.30±0.81		3.32±0.67		3.10±0.84	
6–10	91	22.3	3.19±0.83		2.20±0.82		3.47±0.62		3.02±1.00	
11–20	102	24.9	3.43±0.80		2.31±0.92		3.32±0.86		3.08±0.97	
>20	76	18.6	3.57±0.91		2.40±1.04		3.42±0.78		2.93±0.96	
**Years in this workplace**				<0.001		0.03		0.72		0.26
≤2	159	38.9	3.16±0.82		2.26±0.79		3.40±0.64		3.09±0.91	
3–5	89	21.8	3.40±0.90		2.31±0.90		3.33±0.80		3.17±0.94	
6–10	74	18.1	3.32±0.75		2.15±0.84		3.30±0.78		2.99±0.94	
11–20	74	18.1	3.56±0.78		2.41±0.99		3.42±0.77		2.85±0.97	
>20	13	3.2	3.94±0.99		2.96±1.23		3.53±0.88		3.00±0.71	
**Work shift**				0.29		0.08		0.77		0.82
Diurnal/evening	24	5.9	3.47±0.79		2.51±0.87		3.45±0.75		2.93±0.93	
Night	173	42.3	3.26±0.83		2.19±0.88		3.35±0.78		3.06±0.94	
Mixed	212	51.8	3.38±0.86		2.37±0.89		3.39±0.69		3.04±0.93	
**Employment contract**				0.92		0.78		0.35		0.07
Full-time	212	51.8	3.33±0.87		2.28±0.91		3.40±0.79		2.97±0.98	
Part-time	41	10.0	3.39±0.93		2.27±0.99		3.46±0.73		2.91±0.90	
Contract work	156	38.1	3.33±0.78		2.34±0.83		3.31±0.65		3.18±0.85	

a *P* value obtained by *t*-test or analysis of variance (ANOVA)

SD standard deviation ANOVA analysis of variance F factor

The interviewed personnel reported that there is insufficient staff to manage the patients, that there is not enough emphasis on training of new personnel, and that family medicine OOH trainees are usually not supervised. OOH management was perceived as distant, and a communication gap between service direction and doctors was reported. More than half of the responders (56.8%) reported that they did not receive adequate information about events in the office potentially affecting their work, nor did they receive adequate support for making diagnostic and therapeutic decisions.

## Discussion

### Summary

In this study PSC among Italian OOH doctors was investigated. The findings could be relevant to address the baseline perception of PSC in Italian OOH primary care and target the interventions needed to improve the service.

### Strengths and limitations

Compared to other European experiences, for example a 43% response rate in the Netherlands or 57% in Slovenia,^11,12^ this study had a high response rate (71%).

The application of the SAQ to the OOH population presents several controversies and methodological weaknesses, mainly because findings were limited by the impossibility of analysing all the items. In the present study, 14 items were excluded because >20% of the answers were missing or 'Not applicable'. This is likely because of the Italian context and its work characteristics, which caused several 'Not applicable's, especially in the Teamwork climate factor. This could also have led to a construction of factors with different items from those of the Norwegian^17^ and Dutch^11^ studies.

### Comparison with existing literature

Safety climate has been investigated in Italy in the hospital setting, using different tools and questionnaires,^10,11,18–20^ but never in the OOH primary care setting. PSC has been previously investigated in the OOH setting in Norway, Holland, and Slovenia. Although the content of four factors in the Norwegian,^3,17,21^ Dutch,^11^ Slovenian,^12^ and Italian factor structures bore similarities, several items loaded onto different factors compared to the other models

For comparison to the structure obtained in the other countries, the Norwegian factor structure consisted of five factors and covered 30 items,^17^ the Dutch five factors and 27 items,^11,14^ and the Slovenian five factors and 23 items.^12,22^ The aggregation of factors found in Italy and the characteristics of each factor are probably influenced by the peculiarity that OOH care is provided exclusively by OOH doctors, while in the other systems there is also the involvement of nurses — who were the main responders in the Norwegian^17,21^ and Dutch studies^11^ — and other healthcare professionals.

Notably, even if some factors had the same name across studies, the items included in the factors differ. The Perceptions of Management factor in the Netherlands^11^ and Italy have just four items in common. Some items are in common also with the Dutch^11^ and Norwegian^17^ models, while others were not eligible for factor analysis in the Italian setting, owing to its own characteristics (for example solo working, and having no other healthcare professionals involved in service delivery).

### Implications for research and practice

The OOH doctors work in single-doctor or multiple-doctor OOH centres, without the support of other health professionals. They have basic diagnostic aids and therapeutic equipment. Some are close to or inside hospital or emergency ambulance services, while some are far from such institutions. Although OOH work should be reserved to qualified GPs, some of the doctors are newly qualified and have not specialised, owing to the shortage of GPs. The lack of supervision, scarce engagement of OOH doctors, and the difficulties in management could be overcome with organisational improvement. The educational needs of doctors working in the OOH service should be assessed and met. Mentoring, shadowing, and guiding newcomers to the service would be useful to solve educational needs, while strategies to cope with the impaired communication with staff management, through the implementation of regular dialogue meetings with managers that include audits, briefings, and debriefings at shifts and takeovers, could also improve PSC (that is, provider input).

This study, considering its limitations, shows the need for future research in the OOH settings that encompasses assessment of patient safety, perceptions of management, and even clinical risk in OOH, not to mention the potential early identification of the personnel at risk of burnout. The findings of the survey could be useful to improve OOH GPs' and OOH managers' knowledge of PSC, improve their attitude towards medical errors, and provide interventions, while enhancing consequently the quality of OOH primary care in Italy. Further research with validated tools and strategies to avoid the issue of non-responders and missing answers should inform Italian healthcare decisionmakers in addressing healthcare managers. Interventions on leadership and the development of policies on how managers can improve the safety culture could be effective strategies for the future organisation and efficacy of the OOH service.
